# First and Second Reports of the Commissioners for Enquiring into the State of Large Towns and Populous Districts

**Published:** 1845-10

**Authors:** 


### MEDICO-CHIRURGICAL

### REVIEW.

OCTOBER, 1845.

I. First Report of the Commissioners for inquiring into the State of Large Towns and Populous Districts. Folio, 1844.

Second Report of the Commissioners for inquiring into the State of Large Towns and Populous Districts, with Appendix, Parts 1 and 2. Folio, 1845.

II. A Bill for the Improvement of the Sewerage and Drainage of Towns and Populous Districts, and for Making Provision for an Ample Supply of Water, and for otherwise Promoting the Health and Convenience of the Inhabitants. Prepared and brought in by the *Earl of Lincoln* and Sir *James Graham*. 25th July, 1845.

III. Report of the Surveyor-General of Prisons on the Construction, Ventilation, and Details of Pentonville Prison. 1844.

IV. Contributions to Vital Statistics, being a Development of the Rate of Mortality and the Laws of Sickness from original and extensive data, procured from Friendly Societies, showing the Instability of Friendly Societies, "Odd Fellows," "Rechabites," &c. with an Inquiry into the Influence of Locality on Health. By *F. G. P. Neison*, F.L.S. &c. Actuary to the Medical Invalid and General Life Office. 4to. pp. 140. London, 1845.

V. Pamphlets published by the Health of Towns Association: Abstract of the Proceedings of the Public Meeting; Speech of the Marquis of Normanby in the House of Lords on moving an Address to the Crown on the Sanitary Condition of the People; Suggestions for forming Branch Associations. By *Robert A. Slaney*, Esq.; Unhealthiness of Towns, its Causes, and Remedies. By *R. D. Grainger*, Esq. Lecturer on Physiology at St. Thomas's Hospital.

VI. Letters on the Unhealthy Condition of the Lower

CLASS OF DWELLINGS, ESPECIALLY IN LARGE TOWNS. By the Rev. *Charles Girdlestone*, A.M. Rector of Alderley, Cheshire. 8vo. pp. 92. London, Longman and Co. 1845.

VII. FACTS CONNECTED WITH THE SOCIAL AND SANITARY CONDITION OF THE WORKING CLASSES IN THE CITY OF DUBLIN. By *Thomas Willis*, F.S.S. 8vo. pp. 59. Dublin, J. O'Gorman, 1845.

IT is a painful but unquestionable truth, that so far from the happiness and well-being of the population of England having increased with the daily augmenting wealth of the nation, there has been almost pari passu, a fearful diminution in the material comforts of the people and a corresponding amount of suffering, sickness, and death. We more especially allude to the inhabitants of our manufacturing towns, though in many of the rural districts the population have not escaped this physical degradation. Those who look only on the surface of society and measure the national prosperity by the property of the country as indicated by the increased imports and exports, by the returns of the income tax, and by the general progress of luxury among the higher and even middle ranks of society, may still congratulate themselves that all is well, and that the poor man's hearth shares in some degree in those excessive riches which are at once the envy and the marvel of the world. Would that it were so, and that those who by their unequalled skill and unmatched industry create the country's wealth, reaped in the comfort of themselves and of their families the just reward of their unceasing toil. It is not, however, the first time in the history of the world, that enormous wealth in the hands of the few, has been accompanied with the direst sufferings on the part of the many; nor that unbounded luxury has been seen side by side with squalid wretchedness. The state of things at this time existing in England is powerfully depicted by one thoroughly acquainted with the condition of the manufacturing districts, the Rev. John Clay, of Preston, from whose admirable report we extract the following passage.

> "We endeavour to civilise distant people by winning their confidence, by striving to develop the better qualities of their nature, by promoting intercourse with them, and making them alive to its benefits. The same measures are needed at home, where the moral and intellectual extremes of society are as far asunder as if separated by untrodden deserts or untried seas. This mental remoteness and local propinquity cannot long co-exist without change; a great community is never stationary; there is always a tendency upwards or downwards, according as the few above or the many below exercise influence; while, independent of the movement of the general body, there are ever some individuals sinking, and, happily, more successfully struggling to rise. But the great mass is yet chaotic; and unless, by God's blessing, breathed upon the spirit of intelligence, and of order, and of religion, it may be hurled upon all that is fair and good among us, with a momentum as sudden as irresistible."—*First Report, Ap. Part* 1, p. 55.

To the medical observer, whose duties bring him in immediate and daily contact with the poor and suffering, the sad disclosures made by the successive Commissions, which have investigated the actual condition of our manufacturing population, will only present an aggregate view of that pervading misery, with which, in detail, he has unhappily been so long familiar. But to the influential classes of society in general, it is impossible to conceive of any descriptions more startling, or more calculated to humble the national pride, and to stimulate to national amelioration, than the frightful evils depicted in the Reports and Evidence of the Health of Towns Commission. The curtain which has so long screened from the public eye the almost inconceivable afflictions of the manufacturing poor has at length been raised, and what is presented, not only to the gaze of this country, but to the contemplation of the civilized world:—a population sunk alike in physical suffering and moral debasement; deteriorated in stature, enfeebled by sickness, and fearfully curtailed of the allotted years of human existence. This is strong language, but it is justified by the unparalleled sufferings of hundreds of thousands of our fellow-countrymen, who are doomed in the midst of civilized and Christian England to live a life compared with which the existence of savage and heathen tribes, may, as to material comforts, be termed happy. Can any words, for example, be too forcible to characterize a state of things, in which, out of 100,000 human beings born in Liverpool, in Manchester, or in Birmingham, upwards of 50,000 perish under five years of age; or in which an "artizan reaches only 15 years of age, and has 28 years less chance of life than the gentleman." Well may the inhabitants of the crowded courts and alleys of our great cities exclaim with the inspired writer 'few and evil have been the days of my life.'

The unhappy combination which we have noticed of increasing misery with increasing wealth, is well stated and elucidated by the Marquis of Normanby, in the speech delivered at the formation of the Health of Towns Association. That nobleman, whose strenuous and persevering exertions in the cause of Sanitary improvement are deserving of the highest praise, asks—

"How then did it happen that such a state of things as was exhibited in the reports recently laid before the legislature had been permitted to grow up? His own attention had been first fixed upon the subject in consequence of particular opportunities for observing the details which had occurred to him during the time he had had the honour to hold a high public office. But from that time he had never felt more strongly than at the present moment the conviction, not only that nothing had been done to remove these terrible evils, but that they were actually in a state of daily increasing virulence; and taken, as they must be, in connexion with other circumstances in the social condition of the poorer orders, they gave room for a well-grounded anxiety for the permanence of our national greatness. The most striking instances of the deterioration of physical strength were to be found in the districts where the greatest social changes had taken place within the last thirty years—where the investment of capital and the development of mechanical discoveries had collected a large population together, without an attempt being made to secure their physical well-being. But yet there is scarcely a district exempted from some share in the charge of such neglect. If they turned their glance upon the metropolis, it was not the busy east alone which was obnoxious to it, for in the neighbourhood of the well-ventilated squares of the west there were dense courts and alleys containing within themselves the seeds of disease germinating there, ready to spread their baleful influence around. All this was disgraceful and dangerous."

"The county of Lancaster stood foremost in bad pre-eminence amongst unhealthy counties; but it must be recollected that, in making calculations derived from an extensive district, the comparison was much weakened by there being necessarily a large admixture of a population of a different and sometimes opposite description from that which gave it its peculiar character. In some respects, however, distinct from health, Lancaster had gained greatly; for he saw by the returns upon the last repeal of the income tax, there had been an increase of property in that county as compared with the present time, of 136 per cent. It would be imagined, then, that this was a district in which prosperity most generally prevailed. How was the fact? At the time of the census preceding that repeal of the income tax, the rate of mortality was one in fifty on the whole kingdom. Now the rate of mortality was one in forty-five. How did this happen? How would they account for this diminution of the physical strength of the nation in the face of increased wealth, of improved science in the art of healing diseases, and of improved moral and temperate habits among the people? To solve the difficulty they must point to particular districts of the country, note the statistical facts, and draw their inferences. The mortality in Lancashire thirty years ago was one in forty-six, or less than the general average of England at the present moment. Now the mortality in that county was one in thirty-six. Still there was a difference in the mortality as regarded different districts in the same county. The value of life in Ulverstone, for instance, is stated by Dr. Playfair to be double what it is in Manchester—Manchester which, as he says, enjoys the unenviable notoriety of being the second unhealthy town in the kingdom, and where the mortality was, upon the most favourable estimate, 1 in 28." —*Abstract of Proceedings of the Public Meeting held at Exeter Hall*, p. 6.

We have here disclosed the secret of the apparent anomaly we are considering:—"the investment of capital and the development of mechanical discoveries have collected a large population together, *without an attempt being made to secure their physical well-being*." It is not our wish to hold up the great capitalists and employers of the manufacturing districts to special rebuke; they have only shared in common with the lords of the soil, and we must in justice add, with all the wealthy and influential classes, in that apathetic and selfish indifference to the wants and happiness of their dependents, which is the besetting sin of this utilitarian age. There are, and have been, doubtless, many individual exceptions to this sweeping condemnation, and it is gratifying to know that, since the public attention has been more particularly directed to the existing evils, strenuous efforts have been commenced, and are now in actual progress, in many of the great towns of Lancashire and elsewhere, to obviate the causes of unhealthiness. With these prefatory remarks we proceed to lay before our readers an outline of the deeply interesting matter contained in the various documents now before us.

The most important of these publications are, of course, the Reports and Appendices of the Health of Towns Commission. These comprise three folio volumes or *blue books;* but, in addition to these, and in order to promote the diffusion of their contents among the public generally, an octavo edition has been published, and the reports of the several Commissioners have also been issued as separate pamphlets. The scope of this inquiry will be understood by the following enumeration of the subjects which the Commissioners were instructed to investigate.

"The causes of disease among the inhabitants.

"The best means of promoting and securing the public health, under the operation of the laws and regulations now in force, and the usages at present prevailing with regard to—

"The drainage of lands;

" The erection, drainage, and ventilation of buildings;

" And the supply of water in such towns and districts, whether for purposes of health, or for the better protection of property from fire, and—

" How far the public health, and the condition of the poorer classes of the people of this realm, and the salubrity and safety of their dwellings may be promoted by the amendment of such laws, regulations, and usages." P. vii.

The Commissioners appear to have adopted the most judicious measures for carrying their instructions into operation. They selected for examination fifty towns, in which the rate of mortality appeared by the returns of the registers of death, with a few exceptions, to be the highest. These of course included the largest manufacturing towns and the principal ports, containing a population of more than three millions of persons. A series of questions was transmitted to the municipal and other officers, and subsequently each of these towns was visited by one of the Commissioners, who examined on the spot the general condition of the place, and received statements from medical and other officers, as well as from those among the inhabitants who were capable of affording information. It is satisfactory to find " that in these local inquiries a lively and cordial interest was taken by the inhabitants, and that the Commissioners obtained ready assistance from persons of every class and denomination." We are also happy to perceive that the members of our own profession have not been backward in giving their important aid; as we shall have occasion repeatedly to refer to the evidence, we will only here remark, that the most valuable part of the information collected by the Commission is derived from this source.

In an inquiry embracing such an extended field and such a variety of subjects, we shall select those divisions of it which are of primary importance, or which are more particularly interesting to the medical practitioner; and in doing this, the following order observed by the Commissioners will, for the most part, be followed:

" 1. Drainage, including house and main drainage, and the drainage of any space not covered with houses, yet influencing the health of the inhabitants.

" 2. The paving of public streets, and courts and alleys.

" 3. Cleansing; comprising the removal of all refuse matter not carried off by drainage, and the removal of nuisances.

" 4. A supply of water for public purposes and private use.

" 5. The construction and ventilation of buildings for promoting and securing the health of the inhabitants."—*Second Report,* p. 7.

I. *Drainage.*—The Commissioners state that, " among the evils, which appear to operate with the greatest severity on the condition of all, and especially of the labouring classes, are those arising from the absence of a proper attention to drainage; they prevail almost universally, to an extent altogether incompatible with the maintenance of the public health." And yet, upon this very subject, the public at large, and even many of those more particularly engaged in the matter, architects, surveyors, and builders, entertain the most narrow and mistaken views. As a sample of the ignorance and indifference which has prevailed upon this vital question, it may be stated, incredible as it will appear, that there was till very lately no other than surface drainage in two of the greatest thoroughfares in the metropolis, Bishopsgate Street and Cheapside.

When some of the wealthiest districts in London have thus been strangely neglected, it is not perhaps surprising that the poorer localities, though on account of their crowded population even more urgently requiring efficient sewerage, have been totally abandoned to filth and wretchedness.

The valuable evidence of Dr. Southwood Smith places the evils resulting from the existing state of things, in a strong point of view. Speaking of what he calls *fever districts*, or those parts of the metropolis "in which fever is always so prevalent that the localities in question may be regarded as the ordinary seat of this disease," Dr. Smith observes, "in every district in which fever returns frequently, and prevails extensively, there is uniformly bad sewerage, a bad supply of water, a bad supply of scavengers, and a consequent accumulation of filth; and I have observed this to be so uniformly and generally the case, that I have been accustomed to express the fact in this way:—if you trace down the fever districts on a map, and then compare that map with the map of the Commissioners of Sewers, you will find that, wherever the Commissioners of Sewers have not been, there fever is prevalent; and, on the contrary, wherever they have been, there fever is comparatively absent."—*First Report*, p. 69.

Some details respecting the metropolitan sewers, although laughable from their very absurdity, strikingly illustrate the supineness of the authorities charged with the care of the public health. From the evidence of the Surveyor to the Commission of Sewers for the City, it appears that a certain sum per annum has been expended for the last ten years *in searching out forgotten and unknown sewers*, and among these "lost ones" appears to have been the main sewer in that retired part of London called Smithfield! After such cruel neglect we can hardly be surprised to hear, from the same authority, that one of the sewers "has been exceedingly ill-used, so much so, that, in endeavouring to rout out all the sewers in order to make a plan, we found part of a bedstead in that sewer, and we have even found dead bodies, or at least coffins, in sewers: in one, which passed under a churchyard, we found that it had been used for sepulture, that they had broken into the sewer, and put bodies in it;" nay, by a species of retribution, "they had buried the beadle of the parish in the sewer."—(*L. c.*, p. 209.) It is probably to reward the diligent curators of the City, who are thus meritoriously engaged in redressing the wrongs of their neglected cloacæ, that we find, by the Bill laid before Parliament at the close of the late session, it is proposed, "that this Act shall extend and apply to the whole of England and Wales, *except the City of London and its liberties, and any place situate within a radius of five miles from Charing Cross, in the City of Westminster.*"

If from this graphic picture of the metropolitan sewerage, we turn to other great towns, the same defects are apparent. Many of the streets, courts, and alleys in Liverpool, Manchester, Birmingham, &c., are either entirely without drains, or, if these are provided, they are mere open gutters, adapted only to surface-drainage, and constantly exhaling noxious effluvia. A very intelligent witness largely engaged in building, Samuel Holme, Esq. of Liverpool, states, "there are thousands of houses and hundreds of courts in this town without a single drain of any description; and I never hail any thing with greater delight than I do a violent tempest, or a terrific thunder-storm, accompanied by heavy rain; for these are the only scavengers that thousands have had to cleanse away the impurities and the filth in which they live, or rather, exist."—*L. c., Appendix*, p. 186.

In Manchester it appears that the Paving and Soughing Committee, owing to a want of funds, are only able to carry on their operations in 20 or 30 streets per annum, although there are more than 500 streets in a condition to be proceeded with, and requiring similar amelioration.

Great as are the injurious results of a total absence of sewers, it is a question whether more widely-spread evils do not spring from the manifold and almost universal defects of the sewerage, when it does exist; for, in the one case, the mischief is localised, whilst, in the latter, it operates upon a whole town and upon all its inhabitants. If experience did not show how slow is the progress of improvement, especially when opposed by private and powerful interests, it would have been difficult to comprehend that, in so many of the towns of England, and pre-eminently in the metropolis, there should exist, up to the present moment, a system of sewerage so utterly opposed in all its branches, not merely to scientific principles but to the dictates of common sense.

We need hardly point out to the readers of this Journal, that, for effective drainage, the following are indispensable conditions:—1, That the whole of each natural geological area should be comprised in one plan, and, as a result of this, that all the drains and sewers and pavement within it, should be constructed with reference to each other, as well as to the whole area; 2, that there should be effective means for preventing noxious accumulations in the sewers; 3, that whatever poisonous gases may be disengaged, they should not be permitted to escape near the dwellings of the inhabitants.

Now the evidence shows that there is no city in the kingdom in which all these points are attained; so far from this indeed being the case, it is more usual to find them all neglected.

In describing the impediments existing in London to an efficient system, the Commissioners remark:—

"The drainage of two other districts to the north of the Thames is seriously interrupted by the want of the command over the natural outlets. The Holborn and Finsbury district, which extends over much land that is difficult of drainage, requiring every assistance that engineering science, unfettered by the interference of artificial boundaries, can afford, is cut off from the natural outlet, and is dependent for the means of discharging its waters upon three other districts, the city of London, the Westminster, and the Tower Hamlets divisions. The latter district is again prevented from obtaining the natural outfall by the intervention of the jurisdiction of the Commissioners for Poplar Marsh."

"The inconvenience as well as the enormous loss incurred by these defective arrangements is fully detailed in the evidence contained in the First Report. The sewers within the limits of the City having been laid in at levels, and of a capacity insufficient to convey away the sulliage brought down from the higher district, it became necessary to lower and enlarge those sewers, at an enormous expenditure. Large sums have also been expended in the Holborn and Finsbury district, to take advantage of this improvement. The existence of these evils may in a great degree be traced to the absence of a proper survey, such as we have previously recommended as a necessary preliminary for efficient drainage." *Second Report*, p. 72.

We shall cease to wonder at any difficulties of this kind, when we find that the Metropolis is subdivided into a number of districts, having no reference, with the exception of that south of the Thames, to the natural or geological areas; that, exclusively of the City jurisdiction and that of the Commissioners of Woods and Forests, who superintend the Regent's Park and Regent-street, there are no fewer than five different bodies of Commissioners of Sewers; and, lastly, that the paving and draining of the surface, with the exceptions just stated, are placed under the management of bodies distinct from those which superintend the underground drainage, and for which purpose London "is split up into no less than 84 different jurisdictions, for the government of which at least 129 Acts of Parliament have been passed within the present century."

In the provinces the evils of these conflicting powers are of course experienced in a more limited degree, though in many populous towns, as Liverpool, Manchester, Leeds, Nottingham, Norwich, &c. there are either authorities who thwart each other in their operations, or the jurisdiction is not co-extensive with the natural drainage. The mischiefs arising from this latter circumstance are well exemplified, among other places, at Leeds: "the river Aire, which would in its natural state have had a strong and regular current, had been dammed up in several places for mill-power, and for the purposes of an important water communication. These dams thus act as a series of catch-pits for the sewerage of a population of 120,000 persons. In this case, also, the authorities having control over the town drainage, even if they had been so constituted as to have been competent to execute or maintain systematic works, would have no jurisdiction or control over the natural outfalls; and, in consequence of this original want of jurisdiction and care, rights have apparently been acquired, which can now only be fairly redeemed, for the relief of the town, by purchase."—*Second Report*, p. 17.

With respect to the other conditions we have stated as being essential to a scientific system of drainage, it may be safely averred that, with a few isolated and imperfect exceptions, they are totally disregarded. The existing drains are so constructed as to favour rather than to prevent accumulation of refuse matter; they are, and especially the house-drains, much too large, and are also usually flat at the bottom, two circumstances which, combined with the defective supply of water, lead of necessity to deposits, and these undergoing decomposition, give off those poisonous exhalations, especially sulphuretted hydrogen, which constitute one of the many causes of the destructive diseases which ravage our towns and cities. What other than the most disastrous results can be anticipated when, as we learn from Mr. Roe, the intelligent surveyor of the Holborn and Finsbury district, that foul deposits are allowed to accumulate knee-deep, and remain for two years in a state of fermentation in the sewers beneath the streets; when private drains, opening into our very domiciles, become choked with filth; and when these tubular cess-pools, for such they become, unguarded by effective traps, vomit forth their deleterious gases into every street through the gratings, and into every house through the sinks? The inhabitants of London experience the inconvenience of this wretched system, for there is scarcely a house, even in the wealthiest districts, in which noisome smells are not perceived, especially in the basement story; whilst in the districts to the north of the Thames the stench becomes so offensive that it attracts attention as indicative of a change of weather, whenever strong southerly winds prevail, at which times the air, driving into the sewers debouching in the river, cause a backward current and a consequent escape of gas.

These evils, familiar to all who reside in London, are thus described by Dr. Rigby: "I may mention one family, the members of which were constantly exposed to the effluvia and stench arising from bad drains; they never had a cook remain with them long without suffering severely in health; as the state of the drains became worse they all suffered, and at my urgent advice removed to a healthy suburb of London with the most marked effects in the improvement of health. These defects, as regard effluvia and stench arising from defective drainage, exist also among houses comparatively or quite new: for instance, in the Marylebone district, and even among some of the recently-built houses of Hyde Park."—*First Report*, p. 414.

We are desirous of calling particular attention to the important and interesting evidence of Mr. Dyce Guthrie, a gentleman who has paid great attention to the subject of drainage and the mode of improving it. He remarks, "as the *débris* from any tenement can only be carried off by the water which is furnished to it, the size of the tube to carry off that water after the soil is added to it ought to bear a direct proportion to the supply of water. The soil-pipe of a water-closet is seldom more than two or three inches in diameter, and that pipe never becomes choked up, for the hydraulic pressure is so great that nothing impinging on the interior surface of the tube can resist its force; all matter is regularly washed off each time the tube is flushed, with even the small quantity of water contained in the basin of a common water-closet."—(*First Report*, p 89.) This touches upon a point of infinite consequence, namely, the effectual cleansing of all sewers and drains, an object which, according to the best authorities, can only be accomplished by aqueous agency. It is well observed by a late writer on Geology, Professor Ansted, in alluding to the transporting power of water, that there are certain popular fallacies, concerning the motion of heavy bodies which tend to confuse the judgment; thus it is customary to consider weight as an absolute quality of certain bodies, which are therefore called heavy, though it is, in fact, as usually employed only a relative term. "In this relative sense, a piece of wood is no more heavy when immersed in water, than a balloon filled with hydrogen gas is in the air;" for "in all cases, the actual weight of that quantity of the fluid which would have occupied the space filled by a solid body, must be deducted from the actual weight of the body before the relative weight—the only part which resists motion—can be calculated." It is also necessary to recollect that the power which water possesses of transporting heavy bodies, increases in an enormous ratio with the increased rapidity of the current. Professor Robison has stated, in his Treatise on Rivers, that "a velocity at the bottom of a stream

| | | | |
|---|---|---|---|
| "Of 3 inches | in a second will separate and lift up particles of | .. | fine clay. |
| 6 inches | ditto | ditto .. .. .. .. .. | fine sand. |
| 8 inches | ditto | ditto .. .. .. .. .. | coarse sand. |
| 12 inches | in a second will sweep along and lift up particles of | .. | fine gravel. |
| 24 inches | ditto | ditto .. .. .. | gravel 1 inch diameter. |
| and 36 inches | ditto | ditto .. .. .. | angular stone of the size of an egg. |

"It appears from these experiments, therefore, that a velocity of six inches in a second would be sufficient for scouring away all the usual sediment, and that a velocity of one foot in a second would sweep away fine gravel.

The great merit of applying this transporting power of water to the cleansing of sewers is due to the Commissioners of the Holborn and Finsbury district, who, resting on some interesting experiments made by their intelligent surveyor, Mr. John Roe, have substituted what is called the "*flushing system*," for the old and defective plan of manual labour. This consists essentially in placing dams within the sewers, in certain situations, which collect "heads of water," and this being suddenly set free, carries away all the matter which has accumulated. The great advantages of this method are thus described by Mr. Roe:—

"The great principle intended to be carried out is, that instead of occasional cleansing, as formerly, the sewers should, when once cleansed, be kept free from deposit. The pecuniary saving is, I consider, the least advantage of this mode of cleansing; the great points attained are the avoidance of all accumulations of filth in the sewers, and the stirring up in removal, and consequent disagreeable effluvia, is also avoided; the streets and pavements are undisturbed; the men engaged in cleansing sewers have a more healthy employment than heretofore; private individuals are saved from the annoyance of their drains being choked; and as this plan of flushing affects the health and cleanliness of the inhabitants, the accomplishment of it, on a general and systematic principle, should be deemed of the utmost importance."—*First Report*, p. 134.

Although an ample supply of water is the prime essential in all efficient drainage, yet the form and the dimensions of the sewers are points of importance. The existing sewers, as we have stated, are for the most part flat at the bottom and upright at the sides, the very worst form to prevent deposits and to guard against the pressure of the surrounding earth. The egg-shape, as it is called, has been substituted in the Finsbury district, at Liverpool and other places, and this, by increasing the hydraulic pressure and so lessening the chance of deposits, is doubtless a great improvement. Mr. Dyce Guthrie has, however, suggested what appears to us, and we have conversed with some civil engineers and others qualified to judge upon the subject, a mode of drainage infinitely superior to any of the existing plans. It may be called, from the principle of its construction, *the tubular system of drainage*, the conduits being formed of tile tubes, like those used for field draining, only completely cylindrical. The tubes, which of course vary in size according to the service to be performed, are composed either of common clay or of terra cotta (fire clay); it is further proposed that they should be glazed on the inside, which would secure the immense advantage of rendering the drain *impermeable*, and would thus prevent that leakage of liquid excreta, and that exhalation of poisonous gases which take place in all existing brick-built sewerage. This system would be in various ways very economical, both in original construction and subsequent maintenance; from the nature of the material, terra cotta being selected, it would be very durable, and with a due supply of water it would prevent all accumulation, in fact it would be self-cleansing.

Mr. Guthrie thus embodies the advantages of the tubular system:—

"Your opinion is, that as regards the original construction, for cheapness, for efficiency, and for keeping free from everything deleterious to health, the tubular pipes supplied with water would be the cheapest and best?—Yes; a grand point would be gained by the adoption of tubular drains in the economy of water. Another advantage of my form of house drains would be, the saving of expense effected by the causing the drains of several houses to converge to a point opposite the centre house, at which point only would it be necessary to have an opening into the main sewer. The private drainage of a whole row might in the same manner converge to particular points, at which it would enter the main sewer; by this arrangement one flap or valve would suffice to prevent the escape into the houses of any emanations from the main sewers. The economy of large valves for entrances into the main sewers would of itself be important. I propose, however, a small valve to be placed where it may be thought most convenient on each house drain. Those valves and flaps at present in use are generally objectionable on the score of expense, which arises from their size being unnecessarily great; and their weight, being of metal, must be increased to suit the size, which causes imperfection in their operation. I approve of slate flaps, such as I have seen in the Holborn division of sewers; but I would propose well-burnt plates of common clay, fire clay, or terra cotta, which have the advantage of being readily moulded into the most efficient form. The advantages of flaps of these materials are, that, being of less weight than iron, they are more perfect in their action, and are lifted by a smaller run of water; that they are not affected by chemical action, and therefore are not decomposed, as iron is, by the gases and moisture by which they are surrounded, and that they are originally less expensive than iron, such as those now in use."—*First Report*, p. 93.

The rapidity of construction is another point of great advantage; it would only be necessary to cut a trench the width of the extreme diameter of the tubes, and these "might be placed *in situ* as rapidly as the necessary excavations were completed; you have nothing to do but to place them on the floor of the cut intended for their reception, a vast contrast from the common mode of brick-building, which is a tedious labour." Those of our readers who are well acquainted with the metropolis, can judge of the immense benefit and convenience which would result from any plan that would obviate the constant breaking up of the pavement for the cleansing, repairs and alterations of the sewers; at the time when we write, the two main thoroughfares of London, Holborn and Fleet-street, are both obstructed from these or some similar cause, a state of things which, under a scientific and combined system would be a rare event, instead of, what it literally has become of late years, almost a daily occurrence.

Before quitting this drainage question, we would briefly notice one other point—the imperative necessity of preventing the escape of the poisonous vapours which are so abundantly generated in all sewers as they are now managed. It is apparent that the main improvement would be the effectual cleansing by water; but there are two auxiliary means which will perhaps always, more or less, be required, and which at the present time are quite indispensable, namely, efficient traps and ventilation of sewers. The former contrivances have been alluded to; the latter object is one of difficulty, though it will doubtless be accomplished. One plan is to consume the noxious gases; if the sewers were properly flushed, the water would drive down the gaseous matters, and these might be caught in a receptacle placed at the mouth of the sewer and then burnt; this process has been objected to on the score of the expense. Mr. Guthrie thinks "the most effective mode of ventilating would be to have many small tubes from the crowns of the sewers; these might be laid towards either side of the street, and be carried as high as the tops of house chimneys; the gases would thus be discharged high in the air, and the atmosphere required for respiration would not then be contaminated, as it is at present, by those ventilators which pour forth the gases from the sides or centres of the streets. No mode of ventilating can be more unscientific than the present boasted, though obnoxious system of grated openings in the streets. Tubes, however, might be attached to these, and carried high in all practical localities. Tubes of six or eight inches diameter would be sufficient, and the cost of many thousand yards of these would not equal the expense of a single large chimney-stalk."—*First Report*, p. 99.

II. *Paving*.—Closely connected with the sewerage of a city is the paving of the surface. Those only who are acquainted with the wretched courts, alleys and bye-streets of large towns, can form a just conception of the evils resulting from a want of pavement: the whole surface polluted with filth of every description; mud accumulated in all directions; and, after every shower, water lying in puddles, increasing that excessive moisture which prevails so extensively in the narrow streets and thoroughfares of towns. Even where the streets are paved, owing to a bad system, holes and irregularities exist, catching and retaining impurities of all kinds, and thus adding to the general discomfort.

These statements indicate the imperative necessity of a complete change in the system of drainage, sewerage, and pavement. At present, not only are vast evils inflicted on the health and comfort of the inhabitants, but enormous expenses are incurred, in the payment of officers unnecessarily multiplied, in the provision of premises for the meeting of the Commissioners (the purchase of one house in London for an office, with the cost of repairs, &c. amounted within a fraction to £10,000), in law proceedings, and other less creditable items.

III. *Cleansing and the Removal of Nuisances*.—It is observed by Dr. Arnott, that "the immediate and chief cause of many of the diseases which impair the bodily and mental health of the people, and bring a considerable proportion prematurely to the grave, is the poison of *atmospheric impurity*, arising from the accumulation in and around their dwelling of the decomposing remnants of the substances used for food and in the arts, and of the impurities given out from their own bodies."—(*First Report*, p. 233.) The experience of all medical practitioners will confirm this statement; the contamination of the atmosphere noticed by Dr. Arnott, is in fact the most potential of all the deleterious agencies which are at work in large and crowded cities, for it is through the medium of the respiratory organs that the germs of typhoid, as well as of intermitting and remitting fevers, are introduced into the system. That a large part of this aërial deterioration arises from defective scavenging, and from the multitudinous nuisances of towns, open cess-pools, pigsties, slaughter-houses, &c. is immediately apparent. Now the evidence shows that all these abominations prevail to an extent which is disgraceful to any highly civilised community. From the admirable report of Dr. L. Playfair, on the large towns in Lancashire, it appears that, in Liverpool, Manchester, Rochdale and Preston, the streets are cleansed once in a week; whilst, in other places, Bolton, Wigan, &c. there are no regulations on this important subject.

But it is doubtful if even these rare visits of the scavengers are regularly paid, for Dr. Playfair remarks, " it was my decided impression, from examination of streets in the poorer districts of Liverpool, at intervals of several weeks, that the regulation of the committee as to their cleansing was either not acted up to, or was very inefficiently executed; and in confirmation of the opinion formed from the appearance of the streets, I quote the following passage from the Report by Dr. Duncan, who, himself a native of Liverpool, and intimately acquainted with its localities, has drawn the same conclusion. He says, ' I ought to have mentioned, among the evils requiring remedy in Liverpool, the inefficient system of scavenging and cleansing in the streets inhabited by the poorer classses. The visits of the scavengers to these localities are, I fear, like angels' visits in more respects than one, none of these streets being visited oftener than once a-week, and much longer intervals frequently intervening."—*Appendix, Part* 2, *to Second Report*, p. 12.

These statements refer, it must be recollected, to the paved streets, the rule being that the unpaved and unsewered streets, together with the courts and alleys swarming with inhabitants, and therefore, in an especial manner, requiring careful and repeated cleansing, do not receive any assistance from the public scavengers. The Commissioners observe on this subject:—

" As an illustration of the magnitude of this evil, we may state that in Liverpool there were in the year 1841, 2398 courts, containing 68,365 persons, besides streets, not under the public charge, of which we have no return before us. All these courts, and their numerous inhabitants, are considered to be excluded from the jurisdiction of the Scavenging Committee. Since that time the number of the courts has been increasing, and we regret that we cannot find in the Health of the Town Act, any provision for placing these courts under the same regulations for cleansing, as the other parts of the town. An Act, passed a few days after that here alluded to (July 16, 1842), extends the jurisdiction of the Scavenging Committee over courts, but from the replies received from the authorities, dated September, 1843, it appears that courts are still considered as private property, and cleansed, if at all, only by the occupiers. The same disinclination to exercise any jurisdiction over courts is found to prevail at Leeds. The power contained in the improvement Act in that town for bringing the courts under the jurisdiction of the Town Council, appears to be of little avail, and such places are described to be ' as much neglected as ever.' Nor can we see on what principle of justice, the owners of these places are denied the advantage, which the regulated visits of the scavenger would afford them, since they have been rendered liable to the taxation, which may at any time be imposed for putting their property into good condition. The report, in reference to Birmingham, discloses a similar extent of evil. The courts in the parish of Birmingham, alone are above 2000 in number, and their inhabitants exceed 50,000, besides many in the adjoining parish of Aston."—*Second Report*, p. 37.

London, as usual, forms no exception to the worst state of the worst district in the kingdom. It may be that some of the wretched streets and courts towards the west end are occasionally cleansed by the authorities; but we fear, from the experience of other parts, that the spring of action is rather an anxiety to protect the rich from infectious diseases than any care for the sad sufferings of the poor. Here is a picture, the like of which we have often ourselves seen, taken from the "East-end" of the English metropolis :—

"A short time ago I was standing in one of the streets branching off from Rosemary-lane, called Blue-anchor-yard, looking at a stream of abomination that was flowing down from a court into the open gutter in the centre of this Blue-anchor-yard, the open gutter being the common receptacle for the filth from the houses. This noisome stream was flowing close to a house, at the door of which there stood a woman with ruddy-cheeks neatly clothed. 'Five times this very day, Sir,' said she to me, 'have I swept this place as clean as I possibly could; but you see the state in which it is again. It is no use to try to keep it clean.' Her whole appearance indicated that she was a new comer; in a few days she would naturally give up her hopeless attempt to keep the place clean, and if she remain there she must necessarily sink into the state of squalor and filth, so general among her neighbours."—*Dr. Smith's First Report*, p. 74.

Some interesting details are given as to the comparative amount of debris furnished by the different kinds of pavement. It is stated by Mr. Thorn, a contractor, that the mud on a Macadamised road is three times as much as on ordinary pavement; whilst the accumulation on a wooden road is not more than one-third of that on pavement.

Mr. Whitworth, the inventor of the machine for cleansing streets, and which has been for some time used in a few districts in London, and generally in Manchester, gives some valuable evidence illustrative of the efficiency and economy of this contrivance. He states that at Manchester, he has made an agreement to sweep the streets twice as often as under the old system, and at an actual saving to the town of £500. per annum. Some idea of the efficiency of this plan, which is applicable to every kind of street surface, may be formed from the fact, that whilst a man can on the average sweep not more than 1000 or 1500 square yards daily, the machine worked by one horse sweeps in one hour 4000 square yards, or an average 16,000 to 24,000 per diem; the economy of labour on the whole is so great, that one machine will do the work of 36 men. Mr. Whitworth states that he is engaged in preparing a hand-sweeping machine for courts and alleys, an amelioration which, if properly carried out by the authorities, will be an unspeakable benefit.

Our limits will not allow us even to name the multitude of nuisances which contaminate the air of all large towns, and thus tend to disease. We would, however, call the attention of our readers for a moment to one of the worst evils of this class, the condition, namely, of privies and cess-pools, which constitutes perhaps the greatest discomfort in the lot of the manufacturing poor. The Commissioners observe, with respect to the deficiency of these places, that—

"The extent to which this defect prevails in some of the larger towns is almost inconceivable. At Nottingham it is stated that, under the most favourable circumstances, houses under a rent of 10*l.* have only about one necessary to four or five houses, and frequently the inhabitants of eight or nine houses must resort to one place. In one part of Manchester the wants of upwards of 7000 inhabitants are supplied by 33 necessaries only; and in Ashton, Mr. Coulthart alludes to a locality, where there are only two privies for 50 families. This want of privies is also described as being one of the marked characteristics of the town of Merthyr Tydvil, and in parts even of the Metropolis the deficiency is equally great."—*Second Report*, p. 61.

Not only does this disgraceful state of things lead to every kind of moral and physical debasement, but there is evidence to prove that the noxious exhalations from open cesspools situated close to the houses, are a cause of disease. These circumstances have induced the Commissioners to recommend that, in the case of all new houses, it be made compulsory on landlords to provide proper necessaries for the accommodation of the inmates. We trust, however, that in the progress of improvement cheap and effective water-closets will be generally substituted. It has been proved by a gentleman who has paid considerable attention to the subject, Mr. Austin, that a self-acting water-closet can be supplied and fixed complete for 50 shillings; and when we find that all which is required for draining houses, together with a constant and unlimited supply of water, can be secured for a weekly payment of 2¼d per family, we may anticipate that the above or some similar contrivance, such as those described by Mr. Foden and Mr. Loudon, will be introduced even in the poorest tenements, especially when it is recollected that the expenses of cleansing and maintaining privies and the cost of a miserable supply of water, exceed the weekly estimate above quoted.

IV. *Supply of Water.*—All who are acquainted with the sanitary question are agreed, that without an ample and cheap supply of water, scarcely a single improvement can be effectually carried into operation: this supply is not only required to promote personal and domestic comfort, but likewise for cleansing sewers and the surface of streets and thoroughfares. We are happy to perceive that the Commissioners take a just view of the feelings of the poor on this important subject. They observe that—

"The general and great deficiency in the supplies of water, and the consequent state of filth which the abodes of the poorer classes so constantly exhibit, has, we fear, produced a very general impression, that they are not capable of appreciating the advantages and comfort either of personal or domestic cleanliness. The information derived from the investigations of the Commissioners, and the evidence obtained through other channels, has convinced us that this is a most erroneous view of the feelings and wants of those persons, and we are most desirous to correct this impression, which, if it were well founded, would form a barrier to any prospect of improvement, and would render nugatory the recommendations, that we may subsequently make for facilitating increased supplies of water."—*Second Report,* p. 46.

It is an unquestionable fact that the English are a people pre-eminently distinguished by their cleanly habits; but how is it possible that our humbler brethren can escape from the filth by which they are environed within and without, when the following is a sample of the supply of this prime necessary of life in the crowded parts of London and other great towns:—"the supply of water consists in this: that 16 houses are accommodated with one stand-pipe in the court (Snow's Rents, Westminster.) On the principal cleaning day, Sunday, the water is on for about five minutes, and it is on also for three days in the week for one half-hour, and so great is the rush to obtain a modicum, that perpetual quarrelling and disturbance is the result, and water-day is but another name for dissension." In some crowded districts of London it is stated the poor inhabitants were deprived of water altogether, because the owner of the houses had some quarrel with the water company! A somewhat similar instance lately came to our knowledge of a small landlord, who locked up the only supply of water in a court, whenever any one of his tenants neglected to pay the rent, thus making the whole suffer for the fault or misfortune of one.

The engineer of the Southwark Water Company states, that no less than 5000 tenements in that district, containing 30,000 persons, not being supplied by the Company, "depend for their supplies on pumps or such rain-water as they catch." In many of the manufacturing towns the supply is totally inadequate for their dense population; many of the habitations of the poor have never been properly scoured since they were erected; "many of the poor beg water, many steal it."

The evils of such a system are manifold, and amongst the rest it happens that, the supply being provided often only on alternate days, the water becomes bad and unfitted for use, especially in hot weather. Even when tanks and water-butts are substituted for stand-pipes, the case is not much improved, for these in poor districts are open to the air, and become loaded with dirt, soot, or other impurities, whilst it has been calculated that the expenses entailed on the tenants by these accompaniments of the intermittent system of supply actually amount to one-half of the whole capital invested by the Company; so that "if the Company's capital amount to £50,000. for engines, mains, &c., the tenant's capital invested for tanks, ball-cocks, and pipes, will involve an equal expenditure," the expense of the tank or butt being more than half the tenant's outlay.

Mr. Toynbee gives a painful account of the sufferings caused by this defective supply. This gentleman states—

"I have observed the same water, which is very filthy from having been used in washing some clothes, used again to wash others. They have told me, indeed, that they have done this to avoid the inconvenience of fetching water from a distance, and from the inability to carry the water up stairs when the rooms inhabited have been on the upper floor. My informants on this topic, it should be remembered, are patients, sickly people, weakened by sickness, and who cannot afford to pay for attendance. To the mothers who are debilitated, the carrying water up stairs is a very great exertion: mothers not daring to leave a child in the room, have to carry the child in one arm and the vessel of water with the other. I have had even sick children neglected and left dirty, and the excuse given has been the inability to fetch the water. Recently I have had a case of this kind. I have attended three children, two of them with scrofulous inflammations of the eyes, the other of them with a scrofulous affection of the throat; all of them rarely washed, and in an extremely filthy condition. The mother is a poor woman, who has been in a respectable condition, but she is now so far advanced in pregnancy as to be incapacitated from going up and down stairs to fetch water. She continually deplores her condition of having neither the strength to fetch a sufficient supply of water nor the means of paying for it being brought to her."—*First Report*, p. 340.

This is a picture of thousands of poor families, and which is familiar to every medical practitioner.

We regret that our limits will not allow us to lay before our readers more than a brief notice of the very valuable evidence of Mr. Hawksley, the able engineer of the Trent water-works at Nottingham, respecting the supply of that town by what is called the "high-pressure system:" to this evidence, and that of Mr. Robert Thom, who has devised an efficient plan for the supply of Greenock and other towns, we would direct especial attention. The general features of Mr. Hawksley's method will be understood by a sketch of what he had proposed for the supply of the town of Newcastle-on-Tyne, or of a population of 40,000 or 50,000 people: it must be premised that, in this particular case, the water was to have been taken from a tidal river. A capacious reservoir was to be formed in the clay below the level of low water, into which the water was to be admitted in its best and purest state; "from this reservoir, after depositing the heavier particles, the water was to be raised by a steam-engine, alternately into one of two subsiding tanks, in which tank finer particles of matter held in suspension would be gradually deposited. From this subsiding tank the water would gradually flow upon one of two filter beds, after passing through the materials of which it would be elevated into a reservoir at the head of the town, by means of the same steam-engine by which it was raised into the subsiding tank, and by the same stroke by which other water was being raised into the other subsiding tank. The subsiding tanks, and the filter-beds were to be constructed in duplicate, that one pair might alternately be cleaned, whilst the other pair were in active operation."—*First Report*, p. 310.

At Nottingham, where this improved system has been applied with perfect success for several years, advantage was taken of the nature of the soil, clean sand and gravel, to form a natural filter by the side of the Trent, which at the same time acts as a reservoir, and through which the water slowly percolates for a distance of 150 feet; so perfect is the action that, although the stream is sometimes made so turbid by peat and other vegetable matters that it is of the colour of tea, yet the water after filtration is so bright that a pin may be seen at the depth of eight or nine feet.

Mr. Thom has erected at Greenock, Paisley, and Ayr, *self-cleaning filters*, of a very ingenious construction, and at an expense, for 50,000 inhabitants, of £800. He has also ascertained that "the moss-water, by flowing over or through a particular species of lava or trap-rock (amygdaloid), became fine spring-water." What a contrast do these inventions, by which the water supplied to a whole town is purified by one operation, present when compared with the existing system by which only the comparatively rich can afford to purchase a private filter, which, after all, but imperfectly accomplishes its object.

The greatest pressure at Nottingham is about 120 feet, the average being about 80 feet: this high pressure is kept upon all classes of pipes and at all times, so that the tenants' service pipes, being constantly full and in direct communication with the mains and chief reservoir, the use of tanks and water-butts is entirely discontinued. It is a point of the highest importance that this vast improvement is at once *economical to the inhabitants and remunerative to the company*: thus it appears from Mr. Hawksleys' evidence that, whilst formerly the labouring classes paid for a bucket of water containing three gallons delivered by carriers one farthing, the company supply 79 gallons for the same sum, or, in other words, "delivers water night and day, at every instant of time that it is wanted, at a charge 26 times less than the old delivery by hand." The weekly charge for a house of two or three stories, is about one penny.

The most beneficial results have been produced as to the habits and condition of the people by this unlimited supply introduced into the houses of the labouring-classes; "the increase of personal cleanliness was at first very marked, and was even obvious in the streets; and the medical men reported that the increase of cleanliness was very great in the houses, and that there was less disease."

The advantages of the high-pressure system are not confined to the domestic supply, great as these are; there are other and important benefits which follow in its train. Those who are practically acquainted with the subject, state that the most effectual preservative against fire is to have the mains kept constantly filled with water at high pressure. Mr. Braidwood, for example, the Director of the Fire Brigade in London, being consulted "as to the best mode of protecting the British Museum from fire, advised that that building should be surrounded with mains kept constantly filled with water at high pressure," a recommendation which has been put into operation. At Preston, the superintendent states that, for the last two years, they have never used a fire-engine; the hose being screwed on the plug, the water can be thrown over the highest building, and is more effectual and rapid in its operation. There is no doubt that similar advantages would follow in all places where the plan was fully carried out. At Nottingham, several mills and factories are protected by three-inch pipes, which pass from the main up the stair-case with branches into each room, thus dispensing with the expense of tanks on the roof; an arrangement not only much more economical, but likewise more efficient, for tanks are liable to be soon exhausted, or in Winter frozen, or they may get out of order, which was the case with much of the apparatus kept in the Tower, so that, when required at the late fire, it was found to be unavailable.

The use of water in a sanitary point of view ought also to include the supply of water-closets, the scouring of pavements, the cleansing of carriage-ways, and above all the flushing of drains and sewers, applications which will doubtless in time become universal, as these matters are better understood.

V. *Construction and Ventilation of Streets, Courts, Houses, and Public Buildings.*—This, probably, is the subject to which the attention of medical men has been more particularly directed; at all events it is that in which their co-operation is most desirable, and on which they are most likely to be consulted both by individuals and by the municipal and other authorities; for these reasons it appears desirable to explain the details of ventilation, so far as our limits will permit; we shall also, in this place, allude to some of the more general points of the sanitary question. The reports of Dr. Duncan on the physical causes of the high rate of mortality in Liverpool, of Mr. Holland on Chorlton-upon-Medlock a part of the borough of Manchester, of Mr. Roberton on the amount and causes of death in Manchester, of Dr. Laycock on the sanitary state of York, Dr. Shapter on the state of Exeter, of the Rev. J. Clay on the sanitary condition of Preston, Mr. Coulthart on that of Ashton, and especially the general report of Dr. Lyon Playfair on the large towns of Lancashire, are most valuable documents, containing a vast amount of statistical facts which are deserving of the careful perusal of our professional brethren. The evidence of Dr. S. Smith, Dr. Arnott, Dr. Guy, Dr. Rigby, and Mr. Toynbee is equally important as regards the metropolis.

In considering the causes of the excessive mortality in large towns, it will become apparent that amongst the most influential are the crowded state of the population and the want of a free supply of fresh air.

The most densely populated spot of the empire is unquestionably Liverpool, as we shall see by this Table:—

| Towns. | Inhabitants to Square Mile. | |
|---|---|---|
| | Total Area. | Builded Area. |
| Leeds .. .. .. .. .. .. | 20,892 | 87,256 |
| Metropolis .. .. .. .. .. .. | 27,423 | 50,000 |
| Birmingham .. .. .. .. .. .. | 33,669 | 40,000 |
| Manchester (township) .. .. | 83,224 | 100,000 |
| Liverpool (parish) .. .. .. .. | 100,899 | 138,224 |

Mr. Farr had adduced a small portion of the East of London, containing a population in the ratio of 243,000 inhabitants to a square mile, as the greatest density attained in the heart of English cities; but, according to Dr. Duncan, there is actually a district in Liverpool "containing about 12,000 inhabitants crowded together on a surface of 105,000 square yards, which gives a ratio of 460,000 inhabitants to the geographical square mile; and if we confine the calculation to a smaller portion of this district, but still comprising a population of 8000 (on 49,000 square yards), we shall find the inhabitants packed together in the proportion of 657,963 to the square mile." In Nottingham, which is hemmed in by fields belonging to the freemen, it is stated by Mr. Hawksley that 4,200 people dwell in a square of 220 yards on the side (46,400 square yards), and that the average area to each inhabitant throughout the town, including the streets, is about 18 square yards.

If from whole districts we proceed to inquire into the relative size of the courts and dwellings, the same results are obtained: thus, in a court at Liverpool, with an area of 150 square yards, there is a population of 118 inhabitants, or $1\frac{1}{4}$ square yard to each person; whilst, in those sinks of vice and misery, "lodging-houses," as many as 30 human beings are sometimes packed together in an underground cellar, containing at the utmost 2100 cubic feet. To form some conception how far this horrible crowding tends to the production of disease, it may be as well to add, that the Inspectors of Prisons recommend not less than 1000 cubic feet for every prisoner, as being essential to health and ventilation.

It is generally known that in many towns, principally however confined to Lancashire, though London, Leeds, and other places afford examples, a custom prevails of using cellars as habitations. Without seeing these places it is scarcely possible to conceive of the wretched character of the great majority; they are, as Dr. Playfair terms them, "dismal abodes, badly lighted and worse ventilated." We learn from Dr. Duncan that these cellars are 10 or 12 feet square; that they are generally flagged, though frequently only having the bare earth for a floor; that 1771, or one-third, are from five to six feet deep; 2324 from four to five feet, and 1202 from three to four feet below the level of the streets; 5273, or more than five-sixths, have no windows to the front; and 2429, or about 44 per cent. are reported as being either damp or wet; and lastly, that 1617 have a back-cellar, used as a sleeping apartment, having no direct communication with the external atmosphere, and deriving its scanty supply of light and air solely from the front cellar. The actual condition of the worst of these horrible abodes is sad to contemplate. Mr. Holme, a leading builder at Liverpool, visited one where a poor woman, lately confined, was lying on straw in a vault, through the outer cellar, with a clay floor, and he had to walk on bricks to reach the bed-side, the floor itself being flooded with stagnant water. Dr. Playfair states that, in Clitheroe, "there is a range of cellars, in which the beds are raised on bricks, to keep them out of contact with the water, which, during the periods of much rain, often rises above a foot in depth."

The following is a statement of the number of these wretched abodes and of their population:—

| | Cellars. | Computed Population. |
|---|---|---|
| Liverpool .. .. .. .. | 7,892 | 39,460 |
| Manchester .. . .. | 4,443 | 18,217 |
| Preston .. .. .. .. | 600 | 2,460 |
| Wigan .. .. .. .. | 95 | 276 |
| Bury .. .. .. .. .. | 150 | 615 |
| Rochdale .. .. .. .. | 457 | 1,747 |
| Bolton .. .. .. .. | 1,210 | 4,961 |

Thus there are in this county alone nearly 70,000 human beings living habitually in a condition more wretched than that of the brute creation. The only towns which possess at present any legal powers to deal with this vicious system are Liverpool, Leeds, and London.

It is almost unnecessary to inform medical practitioners that systematic and scientific ventilation is universally neglected both by rich and poor. Dr. Arnott observes: "The subject of ventilation has, as yet, been so little attended to, and is so little popularly understood, that even our Houses of Parliament, in which the intelligent and wealthy of the land assemble, were until lately seriously hurtful to those who had to be much in them from the great impurity of the air occasioned by the crowd. The same fault, unsuspected, exists to a considerable degree in many of our ordinary domestic arrangements; and in workshops and the dwellings of the labouring classes it is one of the great scourges of the people."—(*First Report*, p. 233*). Mr. Toynbee concludes, that defective ventilation is the principal cause of the scrofulous affections which abound amongst dispensary patients; and in the same sense Dr. Duncan quotes the authority of Sir James Clark, who regards "the respiration of a deteriorated atmosphere as one of the most powerful causes of tuberculous cachexia;" and he subsequently adds, "if an infant born in perfect health and of the healthiest parents, be kept in close rooms in which free ventilation and cleanliness are neglected, a few months will often suffice to induce tuberculous cachexia." M. Baudelocque, in his Treatise "*Etudes de la Maladie Scrofuleuse*," adopts the same view, and states that the only cause of scrofula which is met with isolated, or united to circumstances whose action is secondary, is the sojourn, more or less prolonged, in an atmosphere not sufficiently renovated.

There exists of course much diversity of opinion on this point, but no physiologist or practitioner doubts the immense importance of securing an ample supply of fresh air by means of an efficient ventilation. And yet at present, as we have seen, nothing is more neglected; dwelling-houses, especially of the poor, schools, manufactories, churches, work-rooms, all are in a state incompatible with health and comfort. There is no doubt that a large part of the existing evils is to be traced to the repeated failures, which have attended unscientific and blundering attempts at improvement; people have become tired of the trouble and expense which have been thus fruitlessly incurred. We entirely coincide, however, with the Commissioners, who thus express themselves:—"notwithstanding the apparent difficulties with which the ventilation of private dwellings is surrounded, a minute examination of the circumstances of the case has assured us that no field of improvement holds out a more promising result than that which may be anticipated in future from the more successful ventilation, even of the humblest dwellings." We shall return to this subject when considering the remedies suggested for this and the other prevailing defects.

In concluding this general notice of the causes inducing the prevailing unhealthiness of large towns, we are desirous of adding a few words on a point which has latterly attracted considerable attention, the influence, namely, of employments upon health. Dr. Guy, who has investigated this question, especially in reference to the occurrence of phthisis, has obtained some interesting results. Thus, he finds, that sedentary occupations, especially in ill-ventilated apartments, have a greater tendency to produce consumption, than those which are pursued in the open air, and unprotected from the weather. This is shown by the following comparison:—

"Death from consumption under 30:—in-door, 37½ per cent.; tradesmen, 33 per cent.; out-door labourers, 25 per cent.; in-door and sedentary, 44 per cent.; in-door, using great exertion, 31½ per cent."

The proportion of cases in the three classes of gentry (including professional men), tradesmen and artizans (including all classes of labouring men), is respectively, 1 to 5, 1 to 2·60, and 1 to 2·29, or about 16,18, and 30 respectively in the hundred. Dr. Guy believes it to be a vulgar prejudice, that phthisis is more prevalent in England than in other countries.—*First Report*, p. 343.

In a paper read by Mr. Neison before the Statistical Society in the present year and since published, the duration of life in various classes has been, among other matters, carefully considered, the data having been derived from the returns of Friendly Societies in England and Scotland. The general result of these inquiries is, that employment or occupation has even more influence upon health than locality. This view is supported by numerous tables, from which it appears "that, even in the same locality, in the rural districts of the country, where all the supposed contaminating influences of ill-ventilated houses, narrow streets, bad sewerage, poisoned air, epidemic town fevers, and factory restraints, are absent, there is nevertheless a very great superiority in the value of life in one class over another," the great advantage being in favour of agricultural labourers, whilst among painters, weavers, butchers, mill-wrights, bakers, bricklayers, tailors, shoemakers, &c., living in the same rural districts, the value of life is less favourable. The author subsequently observes—

"Some large towns or cities are known to represent a less value of life to their inhabitants generally than other towns; and the explanation usually given of this difference has been the favourable or unfavourable nature of the locality, and a change in the sanitary regulations of the place looked forward to as a certain remedy; but a minute examination of all the external circumstances affecting life will shew, that the great diversity in the mortality of certain classes arises from the influence of other agents. Thus, at age 30, the difference between the expectation of life in the Rural Districts and in Liverpool is 8·2636 years; but the difference between Clerks and Labourers is 13·0211 years; and so also at other periods of life."—*Contributions to Vital Statistics*, p. 56.

The views of Mr. Neison will be sufficiently gathered from the following extracts.

"If this position can be fairly established, it will follow as a direct consequence, that wherever an excess of unhealthy trades are congregated, there must also be an increased rate of mortality independent of the local influence; for if the same trades were placed in any other district, there would still be an increased rate of mortality simply in virtue of the trade or occupation.

"A very small portion of the population in either the Town or City Districts can follow agricultural pursuits; and therefore the standard of life in those Districts will be lowered in consequence of that circumstance alone; but on further examination it will be found that the comparative value of life in those districts is not only lowered in consequence of the absence of many of the most healthy occupations common to the Rural Districts, but that it is still further decreased by the presence of some of the most unhealthy employments, not to be found, or at least to a very limited extent, in the Rural Districts. In other words, the effect of the occupations is such, that if the same people were placed in the Rural Districts, no matter over how much surface they were spread, in order to avoid the influence supposed to connect itself with the congregation of large numbers into towns, still the mortality would be much higher among the people thus conditioned, than among the average of the rural population in ordinary circumstances."—*L. c.* p. 53, 54.

We have made this reference to the work of Mr. Neison, because it places in a prominent point of view, a fact which has always been recognised in a general way, namely, that the nature of the employment will influence the health of the individual who pursues it. But, in according to the author the merit he deserves for the very laborious inquiry he has undertaken, we must express our conviction, that he has entirely underrated the influence of the several causes of disease, which have been considered in the preceding pages; and further, we would observe that the worst results for the public health would arise, if these discouraging notions were adopted as the guide of future action. The limits of this article will not allow us to enter into the details requisite to combat the theory set forth in these contributions to vital statistics, and therefore we must rest content with stating, that we do not perceive how it is possible to reconcile the excessive infantile mortality of cities, which cannot be caused by employment, with the principle advocated by Mr. Neison.

After having devoted so much space to the causes of the existing unhealthiness, we can only more briefly notice the results, and the remedies which have been suggested in the Bill lately presented to Parliament.

The general state of health in a town may be inferred from the mortuary tables, but, in order to apply this test, it is necessary to premise that the mean duration of life for the whole of England in 1841 was 41 years, and the mean age at death 29 years. The cause of the difference in the signification of these terms is not generally understood, and therefore it may be well to quote the following explanatory passage from the Fifth Annual Report (p. 39) of the Registrar-General of Births, Deaths, and Marriages :—" The duration of life in England is 41 years ; if the population were *stationary*, the mean age of those who died would be 41 years, and 1 in 41 would die every year. The population has however increased 1·41 per cent. annually during the last 40 years ; and we find that the mean age of the persons who died in the year 1841 instead of being 41 is 29 years ; while 1 in 46 of the population died."

The following Tables, quoted by Dr. Duncan, give a general view of the relative mortality of London, and the great manufacturing towns :—

| Towns. | Population, 1841. | Deaths. |
|---|---|---|
| Metropolis . . . . | 1,870,727 | 1 in 37·38 |
| Birmingham . . . . | 138,817 | ,, 36·79 |
| Leeds . . . . . . . | 168,667 | ,, 36·73 |
| Sheffield . . . . . | 85,293 | ,, 32·92 |
| Bristol . . . . . . . | 64,298 | ,, 32·38 |
| Manchester (Union) . | 192,408 | ,, 29·64* |
| Liverpool (Parish) . . | 223,054 | ,, 28·75* |

* " The rate of mortality in Liverpool and Manchester is deduced from the average of the five years, 1838-42."

| — | Total Deaths. (two years) 1839-41. | Deaths at 70, and upwards. | Number of Deaths to every 1000 Deaths. | |
|---|---|---|---|---|
| | | | Above 70. | Below 5. |
| Metropolis. . . . . | 93,030 | 10,358 | 111 | 408 |
| Birmingham . . . | 7,426 | 654 | 88 | 482 |
| Leeds . . . . . . . | 8,701 | 688 | 79 | 480 |
| Manchester . . . . | 16,546 | 990 | 60 | 510 |
| Liverpool (Parliamentary) . . | 18,084 | 970 | 54 | 528 |

| | Average Age at Death. |
|---|---|
| " Metropolis, *i.e.* Kensington, Strand, Whitechapel, and Bethnal-Green Unions .. .. .. . .. .. | 26½ years. |
| Leeds .. .. .. .. .. .. .. .. .. .. .. .. | 21 " |
| Manchester .. .. .. .. .. .. .. .. .. .. .. | 20 " |
| Bolton .. .. .. .. .. .. .. .. .. .. .. .. | 19 " |
| Liverpool . . .. .. .. .. .. .. .. .. .. .. | 17 " |

The Table below, compiled from Mr. Chadwick's admirable sanitary report, shows more in detail the influence of the causes of mortality in the several towns enumerated, and on the several ranks of life.

| Towns. | Average Age at Death. | | | General Average. |
|---|---|---|---|---|
| | Gentry and Professional Persons. | Tradesmen. | Labourers, &c. | |
| Kendal . . . . . | 45 years | 39 years | 34 years | 36 years |
| Bath . . . . . . . | 55 " | 37 " | 25 " | 31 " |
| Four Metropolitan Unions | 44 " | 28 " | 22 " | 25 " |
| Leeds . . . . . | 44 " | 27 " | 19 " | 21 " |
| Bolton . . . . . | 34 " | 23 " | 18 " | 19 " |
| Manchester . . . . | 38 " | 20 " | 17 " | 18 " |
| Liverpool . . . . | 35 " | 22 " | 15 " | 17 " |

These documents disclose a fearful amount of mortality in populous districts, especially among those under the age of five years, at which epoch, in fact, more than half of those born are already cut off by death. With such ravages we can understand the observation of one of the medical witnesses, that in the townships of Manchester "the duration of infantile existence needs to be counted by hours or days rather than by months." It is, however, a remarkable and unexpected result of this excessive mortality that, so far from restraining the march of population, it directly and immediately leads to its increase, a circumstance which was first shown by Mr. Chadwick. Mr. Roberton of Manchester has especially illustrated this fact in the following statement, showing the relative proportion of births in healthy and unhealthy districts, and in the different classes of society.

" It appears that the proportion varies from 1 birth in 21·91 persons for the township of Hulme, occupied chiefly by the labouring class, to 1 in 36·48 for Broughton, inhabited generally by the middle and upper classes; and for the town of Manchester, as a whole, 1 in 25·19. With this may be compared the proportion for England, which is 1 in 31; for Wales, and for the metropolis, 1 in 34; for Devon and Hampshire, 1 in 36; and for Salop, which is 1 in 37: a comparison which shows the surpassing fecundity of this manufacturing community."—*Second Report, Appendix,* Part II., p. 107.

Upon this evidence Dr. Playfair remarks:—

" The connexion between excessive births and excessive mortality is, by this time, sufficiently obvious; and careful investigation into facts has brought the indisputable conclusion that disease and pestilence do not always check the increase of our species. Nay—singular and incredible as it may appear, these scourges are not merely powerless to restrain—but they actually give an impulse to—population ! The facts exhibited in the preceding sections will, I apprehend, convincingly show that a crowded and unhealthy district, with all its inevitable accompaniments of low morals and low intelligence,—where the condition of human beings is scarcely above that of animals,—where appetite and instinct occupy the places of higher feelings,—where the barest means of support encourage the most improvident and early marriages,—is not the place where we shall find a diminishing, or even stationary population. For the early unions there are followed by early offspring ; and although more than half that offspring may be swept away by disease during infancy, yet nearly a third of it will grow up, in spite of all the surrounding evils, to follow in the steps of their parents, and, in their turns, to continue a race, ignorant, miserable, and immoral as themselves."—*Second Report, Appendix,* Part II., p. 57.

Such are the results of the morbific agents at work in crowded communities as indicated by the ratio of mortality. There are, however, additional tests, among which the occurrence of fever is the most certain ; for, although other and destructive forms of disease are induced by the malaria of cities, yet typhus may be regarded as a kind of measure of insalubrity, and especially of that which is susceptible of removal by sanitary improvement; the relative frequency of convulsions in infants is another typical index of insalubrity. Dr. Duncan, in noticing the several causes of disease we have been considering, says,—

" But if we are asked to point out more explicitly the *modus operandi* of these causes in increasing the mortality of Liverpool, we reply that they act partly by inducing a specific disease, and partly by deteriorating the general health of the inhabitants in such a way as to render them more prone to the attacks of nearly all diseases, but more particularly of the specific disease alluded to, and also of those organs which feel the first brunt of the poison, *i. e.*, the organs of respiration. The disease I refer to is fever,—the common fever of this country, which may be taken as a generic term, including the varieties known as typhus, synochus, low adynamic fever, brain fever, nervous fever, &c., and which I shall presently show to be the characteristic disease of the poor of Liverpool."—*L. c.*, p. 17.

This excellent observer subsequently remarks :—

" The causes I have mentioned are in operation, more or less, in almost every town, and if it be true that they are influential in the production or extension of fever, we should expect to find that disease more prevalent in towns generally than in the country, where the effluvial gases are more at liberty to follow the bent of their nature, and diffuse themselves through the circumambient atmosphere with velocities inversely proportioned to the square-roots of their densities. And this we find to be the case. Comparing the number of deaths from fever in two years, in the towns and in the country districts comprised in the first table, we find there were 10,930 in the former, and only 6,515 in the latter; the proportion being about 168 in the towns to 100 in the country districts, (which include, however, a number of towns of inferior magnitude.) Further, if it be true that these causes act with greater energy in Liverpool than elsewhere, their effect, or fever, ought also to appear in a greater ratio; and this, too, we find to be the case. Previously to the publication of the Reports of the Registrar-General, I stated my belief, founded chiefly on the records of dispensary practice, that 1 in 25 of the working population of Liverpool was annually affected with fever, and that this probably afforded a higher ratio than any other town in England. This estimate is confirmed by the following table, showing the number of deaths from fever, in the five principal towns of England, during the whole period (3½ years) comprised in the Registrar-General's published reports.

| Towns. | Deaths by Fever.* | Total Deaths. | Per centage Proportion of Fever Deaths to others. | Proportion of Fever Deaths to Population Annually. |
|---|---|---|---|---|
| Birmingham .. .. .. .. .. | 502 | 12,224 | 4·10 | 1 in 917 |
| Leeds (Parliamentary Borough) | 661 | 14,747 | 4·48 | „ 849 |
| Metropolis.. .. .. .. .. .. | 9,150 | 189,379 | 4·83 | „ 690 |
| Manchester (three years).. .. | 1,121 | 19,969 | 5·61 | „ 498 |
| Liverpool and West Derby .. | 2,060 | 33,022 | 6·23 | „ 488 |
| Liverpool (Parish) .. .. .. | 1,795† | 26,456 | 6·78 | „ 407 |

* "Exclusive of scarlet fever and the exanthemata."

† "This gives 513 yearly for the parish alone; and as the mortality of fever in Liverpool is not more than 1 in 12 or 15 cases, 513 deaths will represent about 7000 cases of fever, which, multiplied by 25, will yield more than the estimated number of the working population."—*First Report, Appendix*, p. 19.

The evidence with respect to convulsions is equally significant, the ratio in five of the largest towns in England, being precisely in proportion to the standard of unhealthiness: thus, on an average of the three years, 1838-39-40, the proportion of deaths by convulsions and teething to the total deaths from all diseases was, in Birmingham 5·72 per cent., in London 7·29, in Leeds 12·24, in Manchester 13·69, and in Liverpool 14·93. There can be no doubt that the high state of irritability of the nervous centres indicated by this statement, is essentially caused by the deleterious character of the atmosphere, which attains its utmost intensity among the densely-crowded alleys and cellars of Leeds, Manchester, and Liverpool.

If, from these general indications of unhealthiness, we pass to those more special tests which individualise the causes of disease, the statistical evidence is equally demonstrative. The most searching and discriminative investigation in this point of view is, that of Mr. Holland, who has given a most detailed and interesting account of that part of Manchester called Chorlton-upon-Medlock. In his inquiry, Mr. Holland has separately considered the different classes of streets and houses as to their relative mortality; he has also shown the beneficial results which have followed sanitary improvements, such as the introduction of drainage, pavement, and so-forth. "This analysis shows a very striking difference in the average rate of mortality among the inhabitants of the different streets; for instance, it has been 1·9 per cent. per annum among the inhabitants of the first-class streets and first-class houses, and 4 per cent. in the third or worst-class streets and third-class houses."—*(First Report, Appendix*, p. 63.) A part of this difference of course depends on general causes, poverty, excessive labour, and vicious indulgences; but an extended inquiry still proves that a low rate of mortality has constantly accompanied a good condition of streets and dwellings, and a high rate of mortality the contrary condition.

Dr. Playfair has given a special illustration of this law in the town of Wigan, where it was ascertained that persons following the same occupation in an unhealthy and a healthy district were subject to very different ratios of mortality; that, for example, the tradesman in the former died $4\frac{1}{2}$ years before the tradesman in the latter, whilst in the case of the artisan the difference amounted to $6\frac{3}{4}$ years.

The same results have been observed in all other towns, there being a vast difference in the mortality of the better built parts and those which are over-populated, unsewered, and ill-ventilated: thus the rate of mortality is actually found to be nearly double in some of the neglected districts in the East, compared with what it is in the western part of London.

As it is our object to present a picture of the physical rather than of the moral evils connected with the state of large towns, we shall not enter into any detailed illustration of this latter branch of the inquiry, which indeed to medical men would be superfluous. That a high tone of moral feeling should be sustained in the midst of the squalid misery and material debasement we have described, is a thing all but impossible, and the general tenour of the evidence of the medical and other witnesses confirms this inference. Dr. S. Smith states, with regard to the labouring poor in cities, that as they have not the bodily vigour and the industrious habits of a healthy and independent peasantry, so they have not the intelligence and spirit proper to such a race; "one of the most melancholy proofs of this is the quiet and unresisting manner in which they succumb to the wretchedness of their lot." The reports from Lancashire, where this evil system has attained its acmé, furnish painful proof of the extent to which this moral sickness has infected the town-population. In many of the populous places, in that heart of our manufacturing system, with the ravages of death, and especially with the frightful infantile mortality, there has grown up a class of institutions unknown as to their peculiar features amongst ordinary communities of men, and which are in a marked manner characteristic of those "cities of the plague:" we allude to burial-clubs. In the agricultural districts, it is true, there are sick and benefit clubs, in which allowances are often made on the death of members to defray the funeral expenses; but this is a subordinate, and, as it were, an incidental appendage of those admirable institutions. What, however, is the system as it is at work in Preston? "Most of the sick-clubs," as the Rev. J. Clay states in his excellent report, "are also burial-clubs; that is, a certain sum, varying from £2. to £10., is allowed on the death of a member. There are also in Preston seven societies acting only as burial-clubs, but acting as such very extensively. From the reports sent in, it appears that the present members belonging to three of these societies are upwards of 23,400! (the whole population in 1841 was 50,131); and that the aggregate sum expended by them in funerals, or rather paid to claimants, amounts to £2460. yearly." The worst feature of the case is, that, according to one of the reports, 64 *per cent. of the members who die are under five years of age.*—*1st Rep. Ap.* p. 48.

The evidence further shows that this excessive mortality is made the cloke for pecuniary investments of a somewhat novel character, even in this speculative age. Instances are related in which hired nurses have speculated on the lives of infants committed to their care, by entering them in clubs. Thus, the collector of one of these burial-clubs states, "within these last few days, two young women proposed to him to enter a child into the society, offering to pay the weekly premium alternately; on inquiry he found that the child's mother was dead, and that the infant itself was placed at nurse with the mother of one of these young women." The darkest part of these transactions still remains to be told, for there is good reason to believe that advantage is in some cases taken of another abominable custom, namely, the drugging infants with opium, actually to poison these hapless members for the sake of the funeral allowance; and, as the same person may be entered into several societies, the insurer may receive as much as £16. 6s. 6d., a sum sufficiently large to tempt to the commission of the most heinous crimes, murder itself forming no exception. A case related by Mr. Clay shews that this detestable system invades even that last shrine of human affection, a mother's heart; a lady stated to Mr. Clay, "that a young woman, whose services she required as a wet-nurse, having a child ill, she offered to send her own medical friend to attend it; the reply of the nurse was,—'Oh, never mind ma'am, its in two burial clubs.'"

After such sickening details, it will appear almost an indifferent matter, that the pecuniary penalties inflicted on the community by the excessive mortality and sickness now prevailing, and removable by sanitary measures, are enormous. Our limits will not allow us to enter upon the proof of this position, and we can therefore only state that, according to Dr. Playfair, in the one county of Lancashire alone £5,000,000. per annum are sacrificed in consequence of the attacks of preventible disease! It is also desirable to state, that the greater number of the ameliorations so urgently demanded would in themselves be actually economical. Thus it has been calculated, by one of the most experienced engineers, that an improved and unlimited supply of water may be provided at one-sixth part of the cost of the existing and defective system. Such a supply would further tend to an immense saving in the loss by fire, for it has been ascertained in some of the great cities in the United States, that the cost of insurance has actually been reduced 25 per cent. in consequence of a high pressure being kept up in the mains. By a judicious application of that refuse matter, which in other great towns, after contaminating the air is recklessly wasted, land around Edinburgh has been made to return an annual rent of £30. £40. and even £50. an acre; whilst, in the vicinity of Milan, meadows irrigated by the sewerage water of that city, which in London is allowed to flow into the Thames, are rendered so fertile that they are mowed in November, January, March and April for stable feeding; in June, July, and August, they yield three crops of hay, and again in September they furnish an abundant pasture for cattle.

We cannot better enter upon the consideration of the remedies devised by the Government for the removal of the widely-spread evils we have described, than by quoting the following judicious observations of Dr. Playfair, who, in concluding his excellent report, remarks:—

"That all the facts elicited during the inquiry tend to show that excessive mortality is due to adventitious causes, in almost every instance removable by the combined action of physical improvements, and by the extension of education. Humanity calls loudly for the interference of a paternal legislature to remedy the evils widely spread and deeply rooted—but not irremovable. Sound political economy cannot be in any way opposed to true humanity; and I would say, that all the principles which conduce to the good order and prosperity of the state are involved in the improvement of the sanitary condition of the population." —*L. c.* p. 73.

The full and impartial consideration of the whole subject has forced on our minds a conviction in which we feel assured the public, when sufficiently informed, will entirely participate, that it is vain to expect any general and enduring amelioration without a total change in the whole of the existing system—commissioners of sewers, commissioners of paving, inspectors of nuisances, and other officials as now constituted, must be swept away before any uniform and efficient action can be insured. This indispensable initiative measure, we are happy to find, is provided for by the Bill introduced into the House of Commons at the close of the last Session, "for the improvement of the Sewerage and Drainage of Towns and Populous Districts, and for making provision for an ample supply of water, &c." The hundredth clause of this important Bill is to the following effect:—

"And be it Enacted, That if there shall be, within any town or district made subject to this Act by an Order in Council as aforesaid, any body or bodies of Trustees appointed by or acting under or by virtue of any local Act or Acts of Parliament for the purpose of paving, cleansing, sewering, draining or supplying with water such town or district, or any part thereof, or for any or either of the purposes aforesaid, then on the day to be mentioned in every such Order in Council respectively as the day on which this Act shall come into full force and operation within such town or district, all and every the powers, authorities and duties, of what nature or kind soever, of such Trustees, and of every officer or other person acting under their authority, shall wholly cease and determine."

As a consequence of this provision, the powers and property of every kind possessed by such trustees are to be transferred to the new bodies which are to be appointed for carrying the provisions of the proposed Act into execution.

The Act, as we have already observed, is "to extend to and apply to the whole of England and Wales, except the City of London and its Liberties, and any place situate within a radius of five miles from Charing Cross, in the City of Westminster." It is difficult to conceive upon what principle this exception is made. We have shown that all the evils of the system are as rife in the metropolis as in the provinces; the Commissioners have strongly condemned the multiplication of boards and officers; they have shown the impracticability of carrying out general and enlarged plans of improvements with such conflicting authorities; and they have even found it necessary to make a special report on the drainage and supply of water in London, which they conclude by stating that, "Further legislative enactments are required to improve the laws relating to sewers and the construction of drains to existing houses, to combine the duties of the underground with the surface drainage, to improve the cleansing of small streets, courts, and alleys, and to insure a more liberal and better distributed supply of water."—*Second Report*, p. 75.

As Lord Lincoln, on introducing this Bill had no opportunity of explaining the general views of the Government, it would be premature to pronounce a decided opinion on the subject; but it is certain that no measure which excludes the vast population of London and its suburbs, consisting of more than 2,000,000 *of persons*, from the inestimable advantages derivable from an improved and scientific system, can secure the public approval.

Although there are this and other serious defects in the Sanitary Bill, yet on the whole we are bound to express the highest opinion of its enactments ; it is in fact a sweeping measure of reform, and in such a direction that men of all parties must concur in thanking the Government for the searching inquiry they have instituted, and for the admirable plan they have devised for rectifying the enormous evils and abuses brought to light.

We proceed to lay before our readers the most important provisions of the proposed measure.

All the powers for draining, paving and cleansing towns, for supplying water, removing nuisances, promoting ventilation, &c. are to be vested in each town in one body, consisting of "Commissioners," who are to be nominated in every town as follows: a certain number are to be elected by the occupiers and owners of houses of the rateable value of £10. and upwards, the right of voting being cumulative according to the amount of the property and rental; some are to be elected by the town council, where it exists, out of the aldermen and councillors; a proportion are to be selected by the magistrates who act in borough and other towns, out of their own numbers; and lastly, some are to be elected by the trustees of local acts. The Commissioners, one third of whom are to go out of office every third year, will thus represent the various interests concerned in their proceedings; and although we think the term of office should have been more curtailed, and especially that tenants paying a certain amount of rent should have been made eligible for election as Commissioners, the body constituted as above, promises to be a vast improvement on the existing boards, many of which, especially in London, are essentially self-nominated.

A sufficient number of inspectors "for the purpose of superintending and otherwise assisting in carrying this Act into execution," are to be appointed by one of the Secretaries of State.

One of the best provisions of the Bill is, that which relates to the appointment of "medical officers of health." As it is desirable that our professional brethren, and especially those residing in populous towns and districts, should be acquainted with the nature of these appointments, we append the whole of the two clauses (No. 175 and 176) concerning them.

"175. And whereas the health of the population, especially of the poorer classes, is frequently injured by the prevalence of epidemical and other disorders, and the virulence and extent of such disorders is frequently due and owing to the existence of local causes which are capable of removal, but which have hitherto frequently escaped detection from the want of some experienced person to examine into and repor upon them, it is expedient that power should be given to appoint a duly qualified medical practitioner for that purpose; Be it therefore enacted, That it shall be lawful for the said Commissioners to appoint, subject to the approval of One of Her Majesty's Principal Secretaries of State, a legally-qualified medical practitioner, of skill and experience, to inspect and report periodically on the sanitary condition of any town or district, to ascertain the existence of diseases, more especially epidemics increasing the rates of mortality, and to point out the existence of any nuisances or other local causes which are likely to originate and maintain such diseases and injuriously affect the health of the inhabitants of such town or district, and to take cognizance of the fact of the existence of any contagious disease, and to point out the most efficacious modes for checking or preventing the spread of such diseases, and also to point out the most efficient means for the ventilation of churches, chapels, schools, registered lodging-houses and other public edifices within the said town or district, and to perform any other duties of a like nature which may be required of him; and such person shall be called the Medical Officer of Health for the town or district for which he shall be appointed; and it shall be lawful for the said Commissioners to pay to such officer such salary as shall be approved of by One of Her Majesty's Principal Secretaries of State.

"176. And be it Enacted, That whenever it shall be lawful for any Coroner to summon medical witnesses and to direct the performance of a post-mortem examination, under the provisions of an Act passed in the Session of Parliament held in the sixth and seventh year of the reign of his late Majesty King William the Fourth, intituled, 'An Act to provide for the Attendance and Remuneration of Medical Witnesses at Coroner's Inquests,' it shall be lawful for such Coroner to issue his order for the attendance of the medical officer of health for the town or district within which any such inquest shall be held, and to direct the performance by such medical officer of a post-mortem examination, with or without analysis of the contents of the stomach or intestines, without fee or reward; and any provisions contained in the said Act for imposing any penalty on any medical practitioner for any disobedience of any order of such Coroner shall be taken to extend and apply to such officer of health."

Lastly, the Commissioners are to appoint persons to superintend the due execution of all duties to be performed by the scavengers; to enter in a book all complaints made by any inhabitant of any deficiency in the supply of water, and of any infringement of the provisions of the Act, or of the regulations made by the Commissioners respecting cleanliness and the suppression of nuisances; to visit and examine into the state of slaughter-houses; and to inspect all cattle, fish, &c. intended for food, so as to ascertain if it be unsound or unwholesome: each of such officers is to be called "the Inspector of Nuisances."

Such then, is the *personel* of the Government measure for sanitary improvement. The powers intrusted to the Inspectors and Commissioners are very comprehensive and stringent. In order to secure that essential point in sewerage, uniformity, the Commissioners are to cause a map of the district within their jurisdiction to be provided, on which all that relates to sewers, drains, water-pipes, gas-pipes, &c. is to be marked; the various inequalities of the surface are also to be indicated, and what are called *contour lines,* or lines of equal altitude, are to be inserted. Such a map would be of great service to all parties interested in building and drainage, and in itself must lead to a more scientific mode of procedure.

Authority is given to pave, flag, and put into good order all streets; no new streets are to be made, without the levels having been previously fixed by the surveyor of the Commissioners; all new streets are to be of a certain width, which, for carriage-roads it is proposed shall be not less than 30 feet, and for foot-ways not less than 20 feet. We do not find that any provision is made for regulating the width and size of courts and alleys; and yet if this point is not to be secured, it will be apparent to all who are familiar with the state of large towns, that one of the ameliorations most urgently demanded, will have been omitted.

The part of the Bill relating to sewerage and drainage contains many excellent provisions. The Commissioners are to cause every town subject to the Act, "to be well and effectually sewered and drained," not merely as relates to the public thoroughfares, as at present, but to every house and building within the district. For these purposes power is given to construct sewers where none exist; to provide for properly flushing and cleansing them out; to obviate the injurious effects arising from the noxious exhalations escaping at gully-holes, by providing proper traps, and by the ventilation of sewers, "or by such other ways and means as shall be practicable for that purpose;" to require the owners of all houses, which shall be found not to be properly drained, to construct a covered drain of such materials, size and disposition with respect to fall, as shall be adequate for the drainage of every house, together with its areas, water-closets, privies and offices. Authority is further given to purchase and remove all weirs and mill-dams, causing obstructions to drainage; to compel owners of houses to keep the privies and cess-pools attached to them in good and proper order, according to the regulations of the Commissioners; and further to require the owner of any house to which no sufficient privy or ash-pit is attached, to provide the same in such a manner as shall be requisite for the use of the inmates and occupiers.

By the 167th clause, it is required that all refuse matter, manure, ashes, &c. be removed within a limited period; and, moreover, it is provided, "that if at any time a certificate, signed by the medical officer of health hereinafter authorized to be appointed, or by any *Two* legally qualified medical practitioners, shall be presented to the said Commissioners, certifying that any accumulation of dung, soil or filth within the limits of the said town or district ought to be removed, as being to the prejudice of the health of the inhabitants, the Clerk to the said Commissioners shall forthwith give notice to the owner or reputed owner of such dung, soil or filth, to remove the same within *Twenty-four* hours after such notice." Stagnant pools of water and other annoyances are likewise to be removed.

Ample powers are given for securing to all towns an abundant supply of good water, both for domestic purposes, including baths, the cleansing of house-drains, privies, and water-closets, and "for the efficient and wholesome cleansing of the streets, and the main and other sewers and drains." With this view the Commissioners may purchase or lease the works of existing water-companies; they may also purchase streams of water, construct water-works, lay down pipes, &c.; and, which is a most beneficial provision, they may "erect and place any number of new cisterns, pumps, conduits, or other water-works for the supply of water to the inhabitants of any street, court or alley, or of any number of houses, as they shall see fit, or to erect the same in any public situation, for the gratuitous use of any persons who may choose to carry the same away for their own private use, but not for sale, and to supply with water any public baths or washhouses that may be established for the use of the poorer classes."

In the case of houses, the rent of which does not exceed £10. the Commissioners are empowered to lay down the necessary pipes, and to provide a cistern, &c. for the supply of water. This is another of the excellent features of the proposed Act, but it is clogged with certain conditions, which we trust will be hereafter expunged; we refer to the introduction of this immense improvement being made contingent on the sanction of the owner being first obtained. It is notorious that the persons who own these humble and often miserable tenements, are, as a class, uneducated and frequently impoverished and selfish individuals, who, from these or other causes, are not only indifferent to the wants and comforts of their tenants, but who are even often opposed to all improvement. To leave to such men the power of deciding whether one of the most essential of the sanitary measures shall or shall not be made available in our crowded towns, would not only be in itself a perfect absurdity, but would also, in a multitude of cases, inflict a cruel hardship on our suffering fellow-countrymen.

The Bill contains a clause (No. 189) by which the larger portion of the evils connected with the use of cellars as dwellings will be obviated; for it is therein enacted, that, so soon as the Act comes into operation, "it shall not be lawful to let separately to hire as a dwelling, nor to occupy or suffer to be occupied as such, any room or cellar of which the surface of the floor is more than three feet below the surface of the footway of the nearest street;" there are certain exceptions made to this provision which detract from its value, but it will still be the means of closing a large number of these places.

In order to facilitate the introduction of these various improvements, and, by dividing the expenses incurred, to prevent unfair pecuniary demands being made, in cases where any street, not being a highway repairable by the inhabitants at large, shall have been paved and drained, and when the owner is only tenant for life, or in cases where the amount of the sum to be repaid shall be more than half the amount of the net annual value of the premises, the Commissioners are authorized to allow time for the re-payment of the money expended by instalments of not less than one-twentieth part of the whole sum originally due, interest of course being charged on the part remaining unpaid.

In the preceding remarks and extracts we have summarily noticed all the leading improvements provided for in the proposed Act: there are many other and minor points on which our space will not allow us to comment, but which will be readily understood by the following extract:—

"184. And be it Enacted, That it shall be lawful for the said Commissioners, and they are hereby required from time to time to make bye-laws as they shall think fit, for all or any of the purposes following; (that is to say),

For preventing nuisances and annoyances in any streets, or near thereto, and effecting cleanliness therein:

For making regulations for registering and inspection of slaughter-houses and knackers' yards, and for keeping the same in a cleanly and proper state, and for removing filth therefrom at least *Once* in every *Twenty-four* Hours, and for requiring that they shall be provided with a sufficient supply of water:

For regulating the manner of keeping swine, and preventing the keeping thereof within any dwelling-house, and for describing the limits in such town or district within which it shall be lawful to keep the same:

For the punishment of persons selling unwholesome meat, and for seizing and condemning the same:

For regulating the duties of scavengers, and for regulating the management of public privies:

For making regulations for the registering of lodging-houses, and for maintaining cleanliness therein, and keeping them in a wholesome condition:

For laying down rules for cleansing filthy and unwholesome dwellings."

All the provisions contained in the Act and the duties of the Commissioners, Inspectors, Officers of Health and subordinate employés, are to be enforced by efficient control and penalties, the whole being under the direction of the Secretary of State.

Before dismissing the remedial part of the question, we are desirous of calling attention to the various improvements which have been applied and suggested for promoting one of the most important of the ameliorations demanded, and which to the medical observer is of peculiar interest, the ventilation, namely, of dwelling-houses, public institutions, and all places where persons are congregated together. The works of Dr. Reid, and especially the writings and statements of Dr. Arnott, have made this subject familiar to most professional men. The great objects to be attained are the removal of the vitiated atmosphere, and the maintenance of a medium temperature, without causing draughts, or cold currents of air. Simple as these objects may appear to be, they are, as we have previously noticed, difficult of attainment, and moreover most of the plans, especially when applied on a large scale, are so expensive as to be on that score impracticable.

We may first notice what we deem to be the most urgent case, the ventilation of the small and close dwellings of the poor. For this purpose the balanced valve chimney ventilator, invented by Dr. Arnott, which allows the vitiated air to pass into the chimney, but prevents the return of smoke, may often be applied with great advantage; it is cheap, costing only a few shillings, is most simple in its operation, not liable to derangement, and requires little or no care; indeed it is so efficient, that we feel assured its universal introduction into every sitting-room, drawing-room, and sleeping apartment in the kingdom, whether of rich or poor, would be an incalculable benefit. There are, however, some difficulties opposed to the effective action of this contrivance, the principal of which arises from the defective construction of chimneys, which are, and especially in humble tenements, much too large, and thus prevent the necessary draught. A fire is also requisite to produce a full effect, but this is usually provided, even in Summer, among the poor for domestic purposes. Mr. Toynbee has also extensively applied a ventilating window-pane, which in its simplest form is very cheap, the whole expense being two shillings, and likewise most beneficial in its action. This gentleman, whose extensive experience on the subject entitles his opinion to attention, has no doubt that cheap and effective ventilation might be provided for in every poor man's dwelling, and that it could be enforced in the case of all new buildings, school-rooms, and so forth, without injury; it is therefore much to be regretted, that no express provision is made to secure so desirable an object in the Government measure.

Dr. Arnott has devised a simple and effective plan for ventilating rooms where many persons are collected together, and which might be applied advantageously in a vast number of instances. It is as follows: the skirting-board should be removed from the wall so as to form a space for the admission of fresh air; the space thus obtained, is closed in at the top by finely perforated zinc, and communicates with the external atmosphere by an opening, which, being provided with a valve, regulates the quantity of air to be admitted. The most efficient means for supplying fresh air to large masses of people is, however, the *air-pump*, also contrived by this distinguished and philanthropic member of our profession. The first notion of this apparatus appears to have been derived from the celebrated Dr. Stephen Hales, who proposed a large bellows for the purpose, but which was constructed on such erroneous principles that the value of the invention was greatly diminished. The air-pump of Dr. Arnott drives into the room or place to be ventilated any required quantity of air with a very slight expenditure of power, and it is thus admirably adapted for work-rooms, manufactories, schools, churches, ships, &c.

The evidence of Dr. Rigby, physician to the General Lying-in Hospital, is so valuable, that we are anxious to direct special attention to the subject to which it relates. Dr. Rigby states that, formerly—

"The hospital was seldom free for any length of time from puerperal fever, occasionally producing frightful ravages, and requiring the building every now and then to be closed. After the greatest attention had been paid to cleanliness in every respect, the wards left open night and day for weeks, fumigated, the walls limed and painted, the beds thoroughly cleaned, fumigated, repaired, and frequently renewed, and the most scrupulous attention paid to cleanliness, the fever re-appeared, on some occasions *immediately*, on the hospital being re-opened. This circumstance made us look to external causes, when we ascertained that in the immediate vicinity of the hospital there were upwards of 1500 feet of open ditches, receiving the drainage of the poor and dense population of the neighbourhood, one of the ditches being not more than 30 feet from the walls of the building. It was black and stagnant, and in constant ebullition from the disengagement of gas."—*First Report*, p. 412.

As puerperal fever still continued from time to time, attention was paid to the means of ventilation, with which the physicians were dissatisfied, and with reason, for "the air of the wards was always close, oppressive, and bed-roomy." The hospital was ventilated according to Dr. Reid's plan, and the consequence was, that "the air in the wards became not merely free from effluvia, but has now a remarkably clean, clear, refreshing feel, which I can only compare to the sensation produced on entering an empty room which has been recently whitewashed."—(*L. c.* p. 413.) The most beneficial results followed these improvements; from the moment when the full effects of efficient drainage and ventilation were secured, not a single case of puerperal fever had occurred; "patients have been admitted broken down by poverty and misery; severe and dangerous labours have occurred among them, and there has been every variety of weather, but up to the present time (April 1844) since July there has not occurred the slightest trace of puerperal fever."—*L. c.*

As many of our readers are interested in the ventilation of prisons and jails, we subjoin a brief account of the method adopted at the Model Prison, Pentonville, in which the system of Separate confinement is adopted. The most important point in this and all similar prisons is, of course, the ventilation of the cells, the main objects of which are thus stated:—

"1st. The withdrawal of a stated quantity of Foul air from each cell.

"2nd. The supply of an equal quantity of Fresh air into each cell without subjecting the occupier to the prejudicial effect of a draught.

"3rd. The means of warming the fresh air when necessary, without injuring its qualities or affecting its hygrometrical condition.

"4th. That no additional facilities for the transmission of sound should be afforded by the air-channels or flues."—*Report on the Construction, Ventilation, and Details of Pentonville Prison,* p. 18.

The following is the mode of carrying out these objects. An apparatus for warming the air when required, consisting of a boiler, is placed in the centre of the basement story; from the top of this boiler a rising main communicates, in the usual way, with any number of pipes which may be required for the circulation of hot water, the return-pipes being introduced at the bottom. Around the boiler is an air-flue, into which the external air is admitted and is subsequently conveyed along tubes or flues placed under the floor of the corridors, and from thence passes upwards through small flues preserved in the corridor wall, which terminate respectively in a grating placed close under the arched ceiling of each cell. A grating is placed close to the floor of each cell diagonally opposite to the point where the fresh air is introduced; this grating covers a flue in the outer wall, opening into a horizontal foul-air flue in the roof of the cell, which communicates with a vertical shaft raised 20 or 25 feet above the external roof. The deteriorated air of each cell is withdrawn by the action of this shaft, the air of which is rarified, in the Winter by the fire of the boiler, and in the Summer by a small fire provided for the purpose at the bottom of the shaft: it is found that, if an average difference of 5° to 10° above the external temperature be maintained, the desired effect is produced. From a series of experiments performed by Dr. O. Rees, the principal medical officer of the establishment, the following facts have been determined.

"1st. That from 30 to 45 cubic feet of pure fresh air is made to pass into every cell in a minute; and that the ventilation is maintained with great regularity.

"2nd. That this amount of ventilation, and a temperature ranging from 52° to 60° can be uniformly maintained in the cells during the coldest weather, at an expense of less than a farthing a cell for 24 hours.

"3rd. That the same degree of ventilation is effected during the summer months at less than half that expense."—*L. c.* p. 25.

The general results of this important experiment have doubtless been satisfactory, but we have been informed, by a high authority in these matters, that much still remains to be done to secure complete success. One fundamental error extending throughout the whole of the cells will not have escaped the notice of our readers—the insertion of the grating for the escape of the foul air in the floor, and the introduction of the fresh air at the ceiling. In all improvements of this kind it is essential to bear in mind, that the carbonic acid gas generated by respiration (notwithstanding its intrinsically superior specific gravity) being rarefied by the heat of the body in all warm-blooded animals, becomes lighter than common atmospheric air, and so ascends towards the ceiling, from which, if no escape be provided, it begins to descend, partly by becoming cooler, but principally by diffusion. The same thing also happens in the case of gas-lights, lamps, &c., the heated carbonic acid ascends. In every instance, without exception, in which openings are made for the escape of the products of respiration and combustion, they should, for these reasons, be placed at the upper part of the apartment, or otherwise it is inevitable that the indwellers must breathe a contaminated atmosphere.

The removal of the noxious products of combustion forms an essential part of ventilation in all places where gas-lights and lamps are used; and this has become a point of increased importance in consequence of the general use of gas, not only in shops and public institutions, but in private houses. The simplest and most inexpensive plan is the one now often adopted, to affix, namely, a tube over each burner, carrying this either through the ceiling or roof, or, which is often much better, into the chimney, so as to form a draught of which advantage may be taken to ventilate the room itself. In all these cases, however, it is desirable to make the apparatus which removes the products of combustion, at the same time ventilate the apartment. This has been accomplished by some of the London lamp-makers, and in such a manner as to be ornamental, rather than otherwise; but, as cheapness is in all these matters of primary consequence, we are desirous of noticing a most economical contrivance, described in the report on the state of Exeter by Dr. Shapter. A copper tube, one inch in diameter, is suspended over the glass chimney of an argand gas-burner; the upper part of this tube enters for about one foot into a tube of similar shape but of double the diameter; this larger tube has a wide trumpet mouth, the lip of which is exactly on a level with the ceiling, and can thus receive the vitiated air of the apartment; finally, the upper end of this large tube is carried through the roof, and is protected at the top against the rain by a conical cap. The mode of action is as follows:—"The column of hot air discharged by the glass chimney passes through the smaller tube with great velocity into the larger tube, in which it occasions a rapidly ascending current; whilst, therefore, the smaller tube carries into the larger one all the air vitiated by combustion, the larger tube discharges through the roof, not only the air which is received from the smaller tube, but also the air vitiated by respiration or other causes, which it sucks up from the upper part of the room." This simple and effective apparatus, invented by P. C. De la Garde, Esq., has been in constant use at the Devon and Exeter Institution upwards of seven years, during which time "it has required no cleansing, reparation, or indeed attention of any kind whatever." —*Octavo Report on Exeter*, p. 23.

Our notice of the important matter contained in the Reports and Evidence of the Commissioners of Inquiry has occupied so much space, that we can merely cursorily refer to the remaining works of which the titles are inserted at the head of this article. "The Health of Towns Association" has published some useful tracts with the view of conveying to the public, a knowledge of the main facts elicited by the above-named Commission, and also of preparing the way for those sanitary measures which, as we have endeavoured to show, are so urgently demanded. These pamphlets are well adapted to secure the objects for which they are designed, and we therefore recommend them to the perusal of our readers. Mr. Girdlestone has also published a very useful Abstract of the Evidence, and his "Letters," illustrated by many wood-cuts, may be consulted with great advantage, as presenting a well-digested resumé of the whole question.

It has been our object, in the present article, to lay before our readers a comprehensive view of the bearings of the Sanitary question, which, at this time, engages so large a share of the public attention, and which must of necessity, from the momentous and varied interests concerned, occupy a prominent place in the proceedings of Parliament during the next Session. To our professional brethren more especially the matters we have considered are deeply interesting, and, as many of them will be hereafter called upon to take an important part in the introduction and suggestion of improvements, and as all of them are frequently consulted on the means of obviating the causes of unhealthiness in towns and other populous districts, it becomes no less their interest than their duty, to master the whole subject of which we have, in the present article, notwithstanding the large part of our space we have thought it right to devote for the purpose, only given an outline.

